# CO_2_ Physisorption over an Industrial Molecular Sieve Zeolite: An Experimental and Theoretical Approach

**DOI:** 10.3390/ma16206656

**Published:** 2023-10-11

**Authors:** Anastasios I. Tsiotsias, Amvrosios G. Georgiadis, Nikolaos D. Charisiou, Maria A. Goula

**Affiliations:** Laboratory of Alternative Fuels and Environmental Catalysis (LAFEC), Department of Chemical Engineering, University of Western Macedonia, GR-50100 Kozani, Greece

**Keywords:** CO_2_ capture, industrial zeolite, breakthrough curves, adsorption isotherm, process modelling

## Abstract

The present work studies the adsorption of CO_2_ using a zeolitic industrial molecular sieve (IMS) with a high surface area. The effect of the CO_2_ feed concentration and the adsorption temperature in conjunction with multiple adsorption–desorption cycles was experimentally investigated. To assess the validity of the experimental results, theoretical calculations based on well-established equations were employed and the values of equilibrium, kinetic, and thermodynamic parameters are presented. Three additional column kinetic models were applied to the data obtained experimentally, in order to predict the breakthrough curves and thus facilitate process design. Results showed a negative correlation between temperature and adsorption capacity, indicating that physical adsorption takes place. Theoretical calculations revealed that the Langmuir isotherm, the Bangham kinetic model (i.e., pore diffusion is the rate-determining step), and the Thomas and Yoon–Nelson models were suitable to describe the CO_2_ adsorption process by the IMS. The IMS adsorbent material maintained its high CO_2_ adsorption capacity (>200 mg g^−1^) after multiple adsorption–desorption cycles, showing excellent regenerability and requiring only a mild desorption treatment (200 °C for 15 min) for regeneration.

## 1. Introduction

The removal of CO_2_ from flue gases has gained great significance during the attempt to decarbonize our economy, since the remaining emitters and hard-to-decarbonize sectors face increasing demands to cut down on their CO_2_ output via the integration of carbon capture procedures [1,2,3]. CO_2_ adsorption from solid materials is regarded as an attractive option due to potential energy savings, with solids such as zeolites, activated carbons, and calcium oxides commonly employed for this purpose (including commercially available solid materials) [4]. A CO_2_ adsorbent material is typically required to possess an abundance of surface basic sites with a high affinity towards the mildly acidic CO_2_ molecules [5,6]. Moreover, a high surface area is also desired since it improves the diffusion of CO_2_ molecules and increases the population of available surface sites for adsorption [7,8]. Microporous materials and, more specifically, zeolites are quite popular for CO_2_ capture processes due to their ability to adsorb large quantities of CO_2_ at room temperature, fast uptake kinetics, and regenerability [4,8,9,10,11]. These materials offer a very high surface area and an abundance of CO_2_-philic sites due to the presence of alkaline counterions that charge-balance the negatively charged Al-sites in the zeolitic framework [8,12,13,14]. Moreover, their structural stability allows for reversible CO_2_ desorption at higher temperatures and, as such, they can be used in multiple CO_2_ adsorption–desorption cycles, enabling the cost-effective and efficient capture and release of CO_2_ that helps reduce the energy requirements during the process [4,10,11,15].

Many research works have been published that utilize novel zeolite materials with great performance during low-temperature CO_2_ capture [4,7,8,12,13,16,17]. Indicatively, Dabbawala et al. [7] synthesized a hierarchical porous zeolite Y using a mesopore directing template and managed to achieve a CO_2_ adsorption capacity of approx. 240 mg CO_2_ per g sorbent at 25 °C and 100 kPa, a 20% increase compared to the non-templated zeolite Y. Shen et al. [16] prepared a lamellar LTA-type zeolite from kaolin and showed that the 2D zeolite LTA could capture more CO_2_ than the conventional 3D one, and it also presented a high CO_2_/CH_4_ selectivity. Moreover, Cavallo et al. [13] tested LTA and clinoptilolite zeolites in shaped form for CO_2_ capture at various temperatures. LTA could increase its CO_2_ adsorption capacity following ion-exchange with Ca, while the natural zeolite clinoptilolite could perform better at higher adsorption temperatures, a property that was attributed to its Fe^2+^ ion content inside the zeolitic framework. Typical commercially available zeolites that have been employed for CO_2_ adsorption/separation include ZSM-5, Y (NaY), X (NaX), A (NaA), and other synthetic or natural zeolites [18,19,20,21,22,23]. In general, commercial zeolites are also quite effective for CO_2_ adsorption (for example, 190 mg g^−1^ CO_2_ adsorption capacity was achieved using a commercial 13X type zeolite at 30 °C and 1 bar in Ref. [21]), and they are often employed for comparison purposes in works dealing with laboratory synthetized zeolites [19,22] or in works dealing with modelling of CO_2_ adsorption processes [21,23,24].

The application of theoretical modelling in such adsorption processes is quite important for parameter optimization, and various theoretical models have been previously employed [20,24,25,26,27]. For example, Karka et al. [26] prepared polyethylenimine-modifed zeolite 13x and tested it for CO_2_ capture while also performing kinetic studies. The CO_2_ adsorption kinetics could best be described by the Avrami’s fractional order and the dual kinetic model, while intraparticle diffusion was the rate limiting step. Feng et al. [20] modelled the adsorption isotherms of various gases (CO_2_, CH_4_, CO, N_2_, and H_2_) over a NaY zeolite via the Langmuir, Toth, and Sips equations, while the isosteric heats of adsorption were also calculated via the Clausius–Clapeyron equation. The Toth model was found to be the best fit for CO_2_ adsorption, while CO_2_ demonstrated the highest heat of adsorption when compared to the other gases. Regarding the adsorption of gases other than CO_2_, Georgiadis et al. [27] utilized a commercial LTA-type zeolite with high microporosity, coined as an industrial molecular sieve (IMS), in order to model the room temperature adsorption of H_2_S, and they found that the adsorption results under varying H_2_S feed concentrations could best be fitted by the Langmuir isotherm, whereas the Bangham model could better describe the adsorption kinetics, which were limited by pore diffusion.

In short, zeolite-based materials (commercial, natural, and synthetic) are commonly studied for CO_2_ adsorption because they are considered to be high-performance and cost-efficient adsorbents with high CO_2_ adsorption capacity and fast adsorption kinetics, whereas the temperature of adsorption and efficient adsorbent regeneration are considered some areas that are worthy of further investigation [4,8,9,11]. Furthermore, the combination of experimental adsorption results over commonly available and inexpensive zeolite-based materials with theoretical modelling results is very important, since it allows for the theoretical model validation and parametric optimization of CO_2_ adsorption processes, which is a crucial step towards the design of efficient sorption reactors for practical applications [23,24,27,28].

Based on the excellent adsorption characteristics of the commercial IMS zeolitic material utilized in our previous work [27], we herein employed it again in order to study the physical adsorption of CO_2_ from diluted gas streams and corroborate the experimental results with the ones obtained from computational modelling, taking into account various models and using nonlinear fitting methods (Python). The Python’s curve_fit function that we used herein is an effective tool for curve fitting in many situations and is based on powerful optimization algorithms. Furthermore, contrary to the most frequently used linear methods that may introduce inaccuracies and error propagation, the non-linear approaches employed in this study for the statistical analysis can provide more accurate results. Dynamic adsorption tests (breakthrough experiments) were performed under a varying feed CO_2_ partial pressure and the results were fitted to an adsorption isotherm model. The kinetics and thermodynamics of the adsorption process were also studied under different CO_2_ adsorption temperatures, while three column kinetic models were applied to the experimental data. Finally, the adsorbent was studied under multiple CO_2_ adsorption–desorption cycles, showing the ability to fully regenerate (i.e., recover its initial CO_2_ adsorption capacity) following a mild desorption treatment (200 °C, 15 min). This work therefore contributes to the fundamental understanding of CO_2_ adsorption processes over inexpensive zeolite-type materials, investigates the effect of CO_2_ feed partial pressure and adsorption temperature, and is expected to be useful for researchers working in the field of modelling CO_2_ adsorption processes.

## 2. Materials and Methods

### 2.1. Adsorbent Material

An industrial molecular sieve (IMS) material (Merck) with a zeolitic structure was used for the CO_2_ adsorption tests. The physicochemical properties of this silicon–aluminum and alkali–metallic sorbent are presented in Table 1. Detailed information regarding the structural and textural characteristics and physicochemical properties obtained from X-ray diffraction (XRD), N_2_ adsorption–desorption, and scanning emission microscopy along with energy-dispersive X-ray spectroscopy (SEM-EDX) can be found in Ref. [27]. In short, the zeolite structure corresponds to an LTA-type zeolite (3A or 4A) with well-shaped cubic crystallites (1.5–2 μm). The Si/Al ratio was found to be 0.97 (very close to 1, typical for LTA-type zeolites), while the detected elements by SEM-EDX were Al, Si, Ca, Na, and O, as well as traces of Mg (the majority of counterions were Ca^2+^). The zeolite mostly consists of micropores (possibly also a limited presence of mesopores), displays a Type I N_2_ adsorption–desorption isotherm, and has a specific surface area of approx. 590 m^2^ g^−1^ with an average pore size of 1.73 nm.

### 2.2. CO_2_ Adsorption Tests

**Figure 1:**
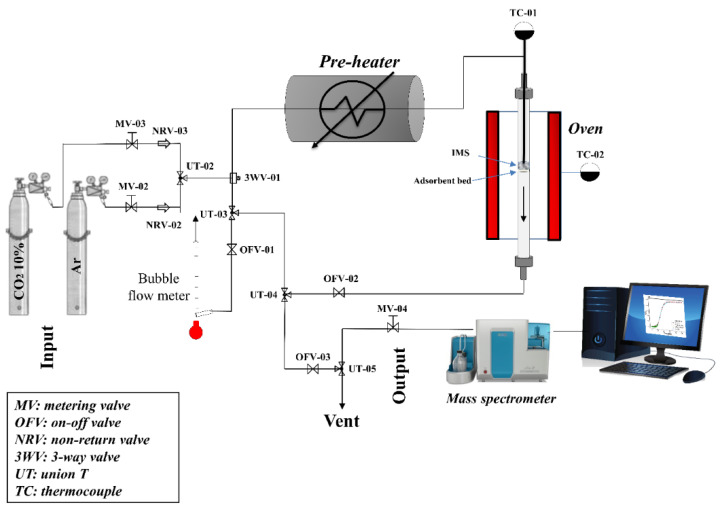
Experimental layout for the CO_2_ adsorption tests.

The dynamic CO_2_ adsorption tests were conducted in a fixed-bed quartz reactor (length: 40 cm, internal diameter: 0.9 cm), which was placed inside an electrical furnace. The schematic of the testing apparatus can be found in Figure 1. The bed height was approximately 2 cm, and it was made by packing 1.0 g of the sorbent material along with inert quartz wool for all the adsorption tests. To measure the temperature, a centered K-type thermocouple was located within the bed. Another K-type thermocouple was responsible for controlling the temperature of the reactor furnace.

The gases used were 10% CO_2_/Ar and Ar (5.0). Gas flows were adjusted by both metering valves (SS) and mass flow controllers. A bubble flowmeter was used to carefully measure the gas flows prior to the initiation of each adsorption test. The analysis was performed on a QMS 300 Prisma mass spectrometer (Pfeiffer vacuum, Aßlar, Germany) to ensure immediate and continuous monitoring for CO_2_ concentrations at the reactor outlet.

To evaluate the CO_2_ adsorption capacity of the IMS adsorbent under atmospheric pressure, numerous CO_2_ breakthrough tests were carried out through altering parameters such as the gas feed CO_2_ volume concentration (i.e., 0.2%, 0.5%, 1%, 2%, 5%, 10%, 20%, 50%, and 100%) and the adsorption temperature (i.e., 25 °C, 35 °C, 50 °C, and 100 °C) while keeping the overall flow rate at 100 mL min^−1^.

Before the experiments commenced, an activation procedure was applied, where the sorbent was pretreated at 300 °C under a flow of Ar in order to ensure that moisture and other adsorbed gaseous impurities (incl. CO_2_) were removed. After cooling down the reactor to the required temperature, a CO_2_/Ar mixture (100 mL min^−1^ total flow rate) was flown through the adsorbent bed. The CO_2_ adsorption capacity was estimated by the following equation [29]:

(1) $$q_{t}=\frac{C_{\mathrm{in}}F_{\mathrm{in}}}{W_{ads}}\int_{0}^{t}\left(1-\frac{C_{t}}{C_{0}}\right)dt$$

where the integral term provides the adsorption time in minutes. In addition, $q_{t}$ (mg g^−1^) is the adsorbed quantity of CO_2_, C_in_ (mg mL^−1^) is the CO_2_ concentration in the bed entry, $F_{in}$ is the total flow rate at the bed entry (mL min^−1^), and $W_{ads}$ is the weight of the adsorbent.

The breakthrough experiments were continued until the value of $C_{t}/C_{0}$ was the one implying the equilibrium of the system, whereas the breakpoint was assumed to occur when the ratio of $C_{t}/C_{0}$ reached the value of 0.05. The regeneration ability of the material was also assessed after undergoing ten adsorption–desorption cycles, with the desorption temperature being 200 °C with a 15 min hold time.

## 3. Results and Discussion

### 3.1. Effect of the CO_2_ Feed Concentration

**Figure 2:**
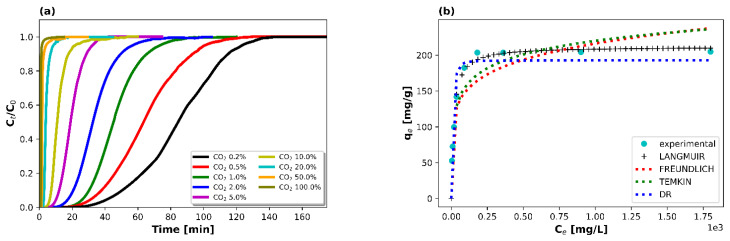
(**a**) CO_2_ breakthrough curves for a gas mixture containing CO_2_/Ar (25 °C, WGHSV = 6000 mL g^−1^ h^−1^) at different CO_2_ feed gas concentrations (i.e., 0.2–10 vol%) and (**b**) predicted adsorption isotherms (i.e., Langmuir, Freundlich, Temkin, DR).

Investigating the effect of CO_2_ feed concentration is crucial for process optimization purposes and the designing of cost-effective and efficient adsorption systems [30,31]. Moreover, different types of flue gases contain a different CO_2_ concentrations (or CO_2_ partial pressures) [32]. As such, a high-performing adsorbent should be able to efficiently capture CO_2_ even at low CO_2_ partial pressures [31,33]. The CO_2_ feed concentration (or pressure) variation also allows us to draw conclusions regarding the CO_2_ adsorption isotherm [7]. That said, the effect of the CO_2_ feed concentration was studied for the IMS molecular sieve in the range of 0.2 vol% to 100 vol% at ambient temperature and pressure with 100 mL min^−1^ of total flow. Figure 2a displays the different concentration profiles tested. In line with the literature, higher CO_2_ concentrations were associated with shorter breakthrough times and steeper breakthrough curves, since the available adsorption sites were saturated faster due to the increased driving force along the pores [27,31,34]. The highest CO_2_ adsorption capacity was recorded at the highest concentration tested (i.e., 205 mg g^−1^ at pure CO_2_ flow), a relatively high value for a zeolite-based adsorbent [8], whilst the lowest one was obtained at 0.2 vol% of CO_2_ (i.e., 53 mg g^−1^). Practically, the isotherm reached a plateau for concentrations values that exceeded 10 vol% of CO_2_ with insignificant changes in the adsorption capacity from that point on (Figure 2b).

In general, a higher CO_2_ feed concentration (or CO_2_ feed partial pressure) can increase the driving force of the CO_2_ gas flow along the pores of the sorbent and thermodynamically favor the CO_2_ adsorption process, which, in turn, can a result in higher CO_2_ uptake values, a behavior that is evident from the steeper breakthrough curves for higher CO_2_ vol% concentrations (i.e., faster and more complete saturation of adsorption active sites) [27,31,34]. Since the adsorption active sites have a finite capacity, it means that they can only adsorb a certain amount of adsorbate molecules before becoming saturated [27,31]. Moreover, from a mathematical point view, when the independent variable (i.e., CO_2_ feed concentration) stops affecting the dependent variable (i.e., CO_2_ adsorption capacity), it suggests that the relationship between the two variables has likely reached a point of equilibrium or saturation. A detailed representation of the results obtained from this series of adsorption tests can be found in Table 2.

Subsequently, in order to optimize the design of the adsorption system by establishing the most suitable correlations for the equilibrium curves, four different adsorption isotherm models (i.e., Langmuir, Freundlich, Temkin, and Dubinin–Radushkevich (DR)), which are typically used for low-temperature adsorption processes, were applied (Figure 2b) [23,27]. Unlike the frequently used linearized forms of these equations, which may increase the propagate errors to the dependent and independent variables, nonlinear methods (Python) were adopted in order to provide more accurate estimations [35,36,37]. In addition, in order to assess the applicability of each model, $R_{adj}^{2}$, which is a modified version of $R^{2}$, was used in order to better express the correlation by also considering the number of independent variables that are added to a particular model. $R_{adj}^{2}$ is given by:

(2) $$R_{adj}^{2}=1-\frac{\left(1-R^{2}\right)\left(n-1\right)}{n-p-1}$$

where $R^{2}$ is the R square, n is the number of rows in the data set, and p is the number of predictors.

The Langmuir equation is defined as [38,39]:

(3) $$q_{e}=\frac{K_{L}q_{max,L}C_{e}}{1+K_{L}C_{e}}$$

where $K_{L}$ is the Langmuir constant, and $q_{max,L}$ is the theoretically estimated CO_2_ uptake. Note that the Langmuir isotherm characteristic form, which is usually obtained when monolayer surface coverage takes place, can also be met in the case of microporous sorbents due to the micropore volume-filling process [40,41].

The Freundlich equation is represented as [38,39]:

(4) $$q_{e}=K_{F}C_{e}^{1/n_{F}}$$

where $K_{F}$ is the Freundlich constant and $n_{F}$ is an indicator of the intensity of adsorption. In particular, a more intense interaction between the adsorbate and the adsorbent can be assumed for higher $n_{F}$ values.

The Temkin equation is expressed as [38,39]:

(5) $$q_{e}=\frac{RT}{B_{T}}ln\left(A_{T}C_{e}\right)$$

where $A_{T}$ corresponds to the equilibrium binding constant, while $B_{T}$ corresponds to the Temkin constant, which is related to the heat of adsorption. The literature mentions that physical adsorption predominates for heat adsorption values that are lower than 20 kJ mol^−1^ [42].

The DR equation is reflected as [38,39]:

(6) $$q_{e}=q_{max,DR}e^{-{\left(K_{DR}RTln\left(1+1/C_{e}\right)\right)}^{2}}$$

where $K_{DR}$ is the DR constant that is related to the mean free energy of the adsorption process, and $q_{max,DR}$ is the CO_2_ adsorption capacity calculated theoretically. As it concerns the mean free adsorption energy, $E_{M}$, it is described as [27]:

(7) $$E_{M}=\frac{1}{{\left(2K_{DR}\right)}^{0.5}}$$

$E_{M}$ values lower that 8 kJ mol^−1^ suggest that the process is governed by physical adsorption phenomena (e.g., van der Waals Forces) whereas for $E_{M}$ values exceeding 16 kJ mol^−1^, chemisorption predominates [27]. Furthermore, $q_{e}$ and $C_{e}$ represent the CO_2_ adsorption capacity and concentration at equilibrium, respectively, in all equations.

The results of the nonlinear fitting are presented in Table 3 and in Figure 2b. Since the Langmuir model demonstrated the highest $R_{adj}^{2}$ value and the theoretically calculated adsorption capacity (211.8 mg g^−1^) diverged only by 3% in comparison to the one obtained experimentally (205 mg g^−1^), it can be assumed that this equation, i.e., Langmuir, can best describe the adsorption isotherm data. The Langmuir isotherm was also found to better describe the CO_2_ adsorption data in other works in the literature [8,16,23,24,40].

### 3.2. Effect of the Adsorption Temperature

Generally, it is known that the adsorption temperature plays a crucial role in the adsorption kinetics, which, in turn, have always been considered to be a significant property of a high-performance adsorbent since the residence time required for the process to be completed, the size of the adsorption bed, and, consequently, the unit capital expenses, are intrinsically related to the rate of adsorption [7,17,27,31,43]. The flue gas temperature is also typically higher than ambient, and thus cooling is often required prior to CO_2_ adsorption [8]. The adsorption temperature variation also allows us to calculate important thermodynamic and kinetic parameters, like the activation energy of the adsorption process, as well as the heat and entropy of adsorption [17,27,43].

**Figure 3:**
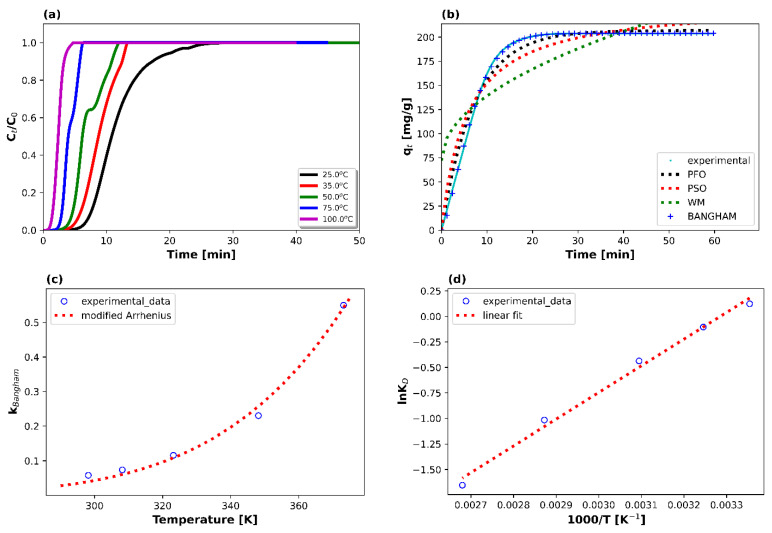
(**a**) CO_2_ breakthrough curves for a gas mixture containing 10 vol% CO_2_/Ar (1 atm, total flow rate = 100 mL min^−1^, WGHSV = 6000 mL g^−1^ h^−1^) at different adsorption temperatures (i.e., 25–100 °C), (**b**) kinetic model (i.e., PFO, PSO, WM, Bangham) fitting for the adsorption data at 25 °C, (**c**) modified Arrhenius equation fitting, and (**d**) thermodynamic parameters estimation.

The adsorption temperature was varied from 25 °C to 100 °C using a feed gas mixture of 10 vol% CO_2_/Ar with the flow being 100 mL min^−1^ (Figure 3a). The observed decreased CO_2_ adsorption capacity values at higher adsorption temperatures are an indication that electrostatic interactions are rather present (and not strong molecular bonding), as is usually the case during low-temperature CO_2_ adsorption processes over zeolitic materials [17,22,31,43]. Typically, increasing the adsorption temperature results in increased molecular velocity and, therefore, weaker electrostatic interactions (e.g., ion–dipole or dipole–dipole interactions) [17,27]. These interactions tend to be less significant as the thermal energy of the molecules overcomes the electrostatic forces, which means that a smaller number of adsorbate molecules can be captured by the adsorbent [17,27,31]. The exothermic nature of CO_2_ adsorption on the zeolitic material (as will be shown later via the negative values of the calculated isosteric heat of adsorption) also means that increasing the adsorption temperature leads to a decrease in the CO_2_ adsorption capacity [43].

Indeed, at 25 °C, the CO_2_ adsorption capacity was calculated at 204 mg g^−1^, whereas at the highest tested temperature (i.e., 100 °C) the adsorbed amount of CO_2_ fell to 35 mg g^−1^, which translates to a decrease of 83%. The CO_2_ adsorption capacities at 35, 50, and 75 °C were calculated at 162, 116, and 65 mg g^−1^, respectively.

In this sense, in order to delve deeper into the adsorption kinetics of CO_2_ by the IMS material, and in order to theoretically calculate the activation energy of the process ($k_{model}$ vs. T), four different kinetic models were applied (i.e., pseudo-first order (PFO), pseudo-second order (PSO), intraparticle diffusion, also known as Weber–Morris (WM), and Bangham).

The PFO equation is written as [44,45]:

(8) $$q_{t}=q_{e,PFO}\left[1-e^{-k_{PFO}t}\right]$$

where $q_{e,PFO}$ is the adsorption capacity calculated theoretically, and $k_{PFO}$ is the rate constant of adsorption for pseudo-first order.

The PSO equation is expressed as:

(9) $$q_{t}=\frac{k_{PSO}q_{e,PSO}^{2}t}{1+k_{PSO}q_{e,PSO}t}$$

where $q_{e,PSO}$ represents the adsorption capacity that the model predicts, and $k_{PSO}$ is the rate constant of adsorption for pseudo-second order.

The WM equation is described as:

(10) $$q_{t}=k_{WM}t^{0.5}+C$$

where $k_{WM}$ is the rate constant of the adsorption process, and C is also a constant.

The Bangham equation is presented as follows:

(11) $$q_{t}=q_{e,B}\left[1-e^{-k_{B}t^{n_{B}}}\right]$$

where $k_{B}$ is the rate constant of the corresponding adsorption process, while n also denotes a constant. The Bangham model typically fits the experimental data when the rate limiting step of the adsorption process is pore diffusion (gas diffusion through the pores) [27,46,47,48]. The term $q_{t}$ in all of the above equation represents the amount of CO_2_ adsorbed at any given time.

The kinetic results obtained from the nonlinear fitting are displayed in Figure 3b and Table 4.

Since the Bangham model exhibits the highest $R_{adj}^{2}$ values, and since the theoretically calculated adsorption capacities, $q_{e,B}$, only deviate by 0.1–0.3% compared to the ones obtained experimentally, it can be said that it best fits the adsorption data and that the process is controlled by pore diffusion. This is in line with other literature works, where microporous materials are used in physical adsorption processes [27,46,47,48].

Afterwards, the rate constants, $k_{B}$, of the best-fitted Bangham model at various temperatures were used to calculate the activation energy of the adsorption process via the nonlinear modified Arrhenius equation (Equation (12)) [27]. The activation energy value was calculated as 32.9 kJ mol^−1^, whilst $R_{adj}^{2}$ was 0.984. This is a relatively low value, in line with other works in the literature regarding CO_2_ physical adsorption [43,49,50].

(12) $$k_{B}=Ae^{-\frac{E_{a}}{R}\left(\frac{1}{T}-\frac{1}{T_{average}}\right)}$$

Thermodynamic parameters, like the heat and the entropy of adsorption, were then calculated through the Van’t Hoff equation. The linear equation used is the following [37]:

(13) $$lnK_{D}=-\frac{\Delta H^{o}}{RT}+\frac{\Delta S^{o}}{R}$$

where $K_{D}$ is defined as the ratio $q_{e}/C_{e}$.

The slope and the intercept of the above equation provide the values of adsorption enthalpy and adsorption entropy, respectively, given that the high estimated $R_{adj}^{2}$ value (i.e., 0.992) ensures the linear relationship between the dependent and the independent variables. In particular, the calculated values were −21.8 kJ mol^−1^ for the heat of adsorption, $\Delta H^{o}$, and −71.5 J mol^−1^ K^−1^ for the entropy of adsorption, $\Delta S^{o}$. This corroborates, on the one hand, the exothermic nature of the physical adsorption process ($\Delta H^{o}$ < 0) and, on the other hand, the insignificant internal structural changes that the material undergoes during the process. These results seem to be in agreement with those of other literature works, where it is reported that the absolute $\Delta H^{o}$ values in physical adsorption processes lie between 20 and 45 kJ mol^−1^ [17,43,51].

### 3.3. Column Studies

Three column kinetic models, Adams–Bohart, Thomas and Yoon–Nelson, were then applied to the data obtained experimentally for the prediction of the breakthrough curves using nonlinear methods in order to determine those parameters that are characteristic of the column and that are necessary for the process design of the sorption reactor.

The Adams–Bohart (AB) model is described as [52,53,54]:

(14) $$\frac{C_{t}}{C_{0}}=exp\left(k_{AB}C_{0}t-k_{AB}N_{0}\left(\frac{h}{v}\right)\right)$$

where $C_{0}$, v, and h are the initial concentration, initial flow velocity, and bed height, respectively, whereas $k_{AB}$ is a rate constant, and $N_{0}$ is the saturation concentration. To describe the initial part of the breakthrough curve ($C_{t}/C_{0}<0.15$), this model is quite suitable. It postulates that the adsorption rate is proportional to both the adsorbing species concentration as well as the residual adsorbent capacity.

The Thomas (TH) model is expressed as [53,54]:

(15) $$\frac{C_{t}}{C_{0}}=\frac{1}{1+exp\left(k_{TH}q_{e,TH}\frac{W}{Q}-k_{TH}C_{0}t\right)}$$

where $C_{0}$, Q, and W are the initial concentration, initial flow rate, and weight of the adsorbent, respectively, whilst $k_{TH}$ is a rate constant, and $q_{e,TH}$ is the adsorbate quantity that is adsorbed by the adsorbent. Principally, this model is used to predict the maximum adsorption capacity of an adsorbent and is required for the design of a bed column.

The Yoon–Nelson (YN) model is written as [54,55]:

(16) $$\frac{C_{t}}{C_{0}}=\frac{exp\left(k_{YN}\left(t-\tau \right)\right)}{1+exp\left(k_{YN}\left(t-\tau \right)\right)}$$

where $k_{YN}$ is the adsorption dynamic constant, and τ corresponds to the time required for retaining 50% of the initial adsorbate concentration. The advantage of this model lies in its simplicity, given that no detailed data with respect to the adsorbate characteristics, the physical properties of the adsorption column, and the type of the adsorbent are needed. The Yoon–Nelson equation postulates that the rate that the probability of adsorption for each adsorbed species decreases is proportional to the adsorbate breakthrough probability on the adsorbent and the probability of adsorbate retention.

**Figure 4:**
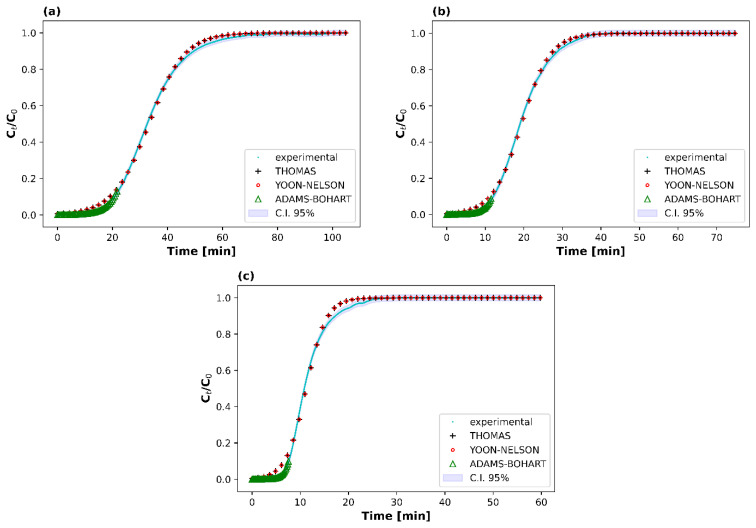
Model breakthrough curves for different initial CO_2_ feed concentrations: (**a**) 2 vol%, (**b**) 5 vol%, and (**c**) 10 vol% (25 °C, 1 atm, total flow rate = 100 mL min^−1^, WGHSV = 6000 mL g^−1^ h^−1^).

The obtained column data were fitted to the three aforementioned equations (Equations (14)–(16)) using nonlinear methods, and the results are presented in Table 5 and Figure 4. The determined $R_{adj}^{2}$ range from 0.992 to 0.999 indicates a significant correlation between $C_{t}/C_{0}$ and t.

By comparing the values of $R_{adj}^{2}$, it seems that the models of Thomas and Yoon–Nelson better describe the adsorption behavior. Interestingly, the Thomas model predicted the CO_2_ adsorption capacity of the IMS adsorbent with excellent precision in the case of 5 vol% and 10 vol% CO_2_ feed concentration, presenting less than 5% deviation between the theoretically and the experimentally obtained values for the equilibrium CO_2_ adsorption capacity. The predicted CO_2_ adsorption capacity diverged by 13% when 2 vol% of inlet CO_2_ concentration was used, which is again a fairly good prediction. Regarding the Yoon–Nelson model, it can be observed that the rate constant, $k_{YN}$, was increased and the 50% breakthrough time, τ, was decreased upon increasing the inlet CO_2_ concentration, as anticipated, meaning that a higher CO_2_ feed concentration benefits the adsorption kinetics [31]. A good fitting to the aforementioned models has also been reported in other literature works regarding CO_2_ adsorption [54,56].

### 3.4. Effect of Multiple Adsorption–Desorption Cycles

**Figure 5:**
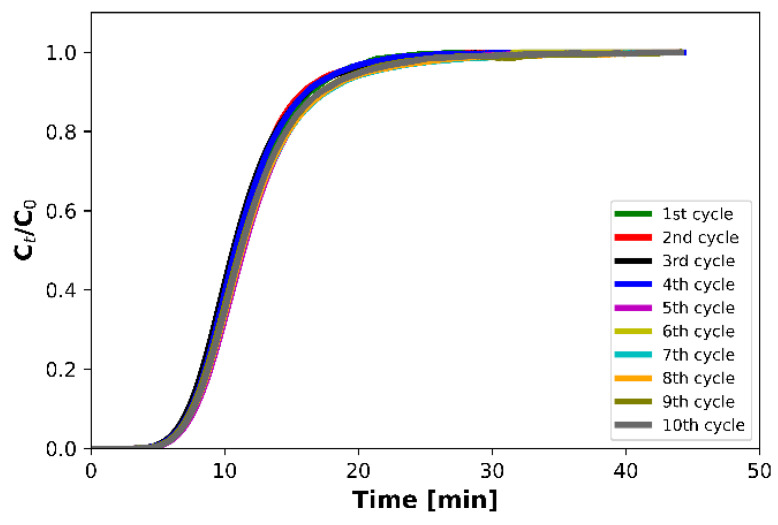
CO_2_ breakthrough curves during 10 adsorption–desorption cycles (Adsorption: 25 °C, 10% CO_2_/Ar, 1 atm, total flow rate = 100 mL min^−1^, time = 45 min, WGHSV = 6000 mL g^−1^ h^−1^; Desorption: 200 °C, Ar, 1 atm, total flow rate = 100 mL min^−1^, time = 15 min).

Cyclic experiments of adsorption followed by desorption were also conducted for a total of 10 cycles in order to study the stability and regenerability of the IMS adsorbent (Figure 5). The experimental conditions were 10% of inlet CO_2_ volume concentration in Ar, ambient temperature and pressure, and 100 mL min^−1^ of total flow. The activation procedure was carried out once at 300 °C prior to the first cycle. Desorption was carried out after each cycle at 200 °C for 15 min under pure Ar flow.

The results revealed that the CO_2_ adsorption capacity was only slightly altered during the repeated CO_2_ adsorption–desorption cycles and was kept in the range between 200 and 205 mg g^−1^ (Table 6), with the small discrepancies between the calculated values being within the experimental error. The difference between the highest and the lowest adsorption capacity value was just 2.1%. As such, great reproducibility of the repeated experiments was ensured by the fact that the obtained values laid well within the corresponding confidence intervals, meaning that the adsorption process is fully reversible.

In general, the mechanism of this reversible CO_2_ adsorption process is expected to proceed via the formation of linear CO_2_ molecular species and probably also some weakly-bound and labile carbonate-like complexes in the cavities of the zeolite [57,58]. According to Martin-Calvo et al. [58], the presence of more labile and easy-to-desorb carbonates agrees with the fact that the majority of the alkaline counterions in our zeolite are bivalent calcium ones (according to the SEM-EDX analysis reported in Ref. [27]). This suggested adsorption mechanism corroborates the reversibility of the CO_2_ adsorption process, as these weakly-adsorbed linear molecular species and/or labile carbonate-like complexes are apparently completely removed following the desorption treatment [19,31]. The process reversibility is also supported by the theoretical calculations, where low values of activation energy and enthalpy of adsorption were found, since, generally, the activation energy and adsorption enthalpy values are indicators of the interaction strength between the adsorbate molecules and the solid adsorbent [43,51,59]. This very high reversibility/stability also means that the desorption treatment does not cause any detrimental structural modifications on the adsorbent material and that it can be used in thermal swing adsorption processes [19,31].

## 4. Conclusions

In the work presented herein, a commercial LTA-type zeolite material, coined as IMS, was employed during the study of CO_2_ adsorption. A joint experimental and theoretical approach was adopted to gain insight into the equilibrium, kinetic, thermodynamic, and column design properties of the CO_2_ adsorption process. The key results are synopsized below:

- By varying the initial CO_2_ feed concentration (or partial pressure), it was found that a higher feed concentration led to increased CO_2_ adsorption capacity. The CO_2_ adsorption isotherm could best be fitted to a Langmuir type isotherm and reached a plateau for CO_2_ feed concentrations that exceeded the value of 10 vol%.
- By varying the CO_2_ adsorption temperature, it was found that a higher temperature led to decreased CO_2_ adsorption capacity. This is indicative that CO_2_ is adsorbed via van der Waals forces, or, rather, via electrostatic interactions (i.e., physical adsorption).
- The Bangham model (i.e., pore diffusion) could best describe the adsorption kinetic behavior, as is usually the case in physical adsorption processes.
- The activation energy (E_a_) was calculated at 32.9 kJ mol^−1^ and the adsorption enthalpy (ΔH^o^) at −21.8 kJ mol^−1^, indicating that CO_2_ adsorption over the IMS adsorbent is exothermic.
- The Thomas and Yoon–Nelson kinetic models were the most suitable to describe the adsorption process of CO_2_ on the IMS adsorbent during column studies.
- Lastly, the IMS adsorbent successfully maintained its high adsorption capacity (>200 mg g^−1^) after 10 consecutive adsorption–desorption cycles, with desorption/regeneration being carried out under mild conditions (200 °C for 15 min).

## Figures and Tables

**Table 1:** Characteristics of the IMS adsorbent.

Characteristic	Value
Shape ^a^	Spherical
Density (g dm^−3^) ^a^	1.36
Bulk density (kg m^−3^) ^a^	700–750
pH ^a^	8–11
Melting point ^a^	<1600 °C
Si/Al ratio ^b^	0.97
Specific surface area (m^2^ g^−1^) ^c^	590
Average pore size (nm) ^b^	1.73

^a^ Obtained from the manufacturer’s product specification sheet. ^b^ Obtained from SEM-EDX analysis. ^c^ Obtained from N_2_ adsorption–desorption analysis.

**Table 2:** Effect of CO_2_ feed concentration on the equilibrium adsorption capacity.

CO_2_ Feed Concentration (%)	Equilibrium Adsorption Capacity (mg g^−1^)
0.2	53
0.5	73
1	100
2	142
5	183
10	204
20	204
50	204
100	205

**Table 3:** Theoretically estimated parameters of the Langmuir, Freundlich, Temkin, and DR models.

Model	Parameter	Value
Langmuir	$K_{L}\left(L\;{mg}^{-1}\right)$	0.06
	$q_{max,L}\left(mg\;g^{-1}\right)$	211.8
	$R_{adj}^{2}$	0.979
Freundlich	$K_{F}\left({mg}^{1-1/n_{F}}g^{-1}L^{1/n_{F}}\right)$	71.66
	$n_{F}$	6.26
	$R_{adj}^{2}$	0.704
Temkin	$A_{T}\left(L\;{mg}^{-1}\right)$	2.98
	$B_{T}\left(kJ\;{mol}^{-1}\right)$	0.09
	$R_{adj}^{2}$	0.836
DR	$K_{DR}$	2.3 × 10^−5^
	$q_{max,DR}\left(mg\;g^{-1}\right)$	192.6
	$E_{M}(kJ\;{mol}^{-1})$	147.6
	$R_{adj}^{2}$	0.721

**Table 4:** Theoretically estimated kinetic parameters for PFO, PSO, WM, and Bangham kinetic models.

Model	Parameter	25 °C	35 °C	50 °C	75 °C	100 °C
PFO	$k_{PFO}\left({min}^{-1}\right)$	0.139	0.191	0.234	0.411	0.714
	$q_{e,PFO}\left(mg\;g^{-1}\right)$	206.92	164.40	117.51	65.66	34.59
	$q_{e,exp}\left(mg\;g^{-1}\right)$	203.69	162.23	116.28	65.14	34.46
	$R_{adj}^{2}$	0.979	0.970	0.977	0.970	0.979
PSO	$k_{PSO}\left({g\;{mg}^{-1}min}^{-1}\right)$	7.7 × 10^−4^	1.5 × 10^−3^	2.7 × 10^−3^	1.0 × 10^−2^	3.8 × 10^−2^
	$q_{e,PSO}\left(mg\;g^{-1}\right)$	235.78	182.56	129.11	69.83	36.06
	$q_{e,exp}\left(mg\;g^{-1}\right)$	203.69	162.23	116.28	65.14	34.46
	$R_{adj}^{2}$	0.927	0.901	0.908	0.877	0.871
WM	$k_{WM}\left({mg}^{-1}\;g^{-1}{min}^{-1}\right)$	21.18	15.11	10.52	4.38	1.73
	$C\;\left(mg\;g^{-1}\right)$	72.05	73.51	57.57	42.40	26.06
	$R_{adj}^{2}$	0.650	0.552	0.528	0.375	0.277
Bangham	$k_{B}\left({min}^{-n}\right)$	0.058	0.074	0.116	0.230	0.550
	$q_{e,B}\left(mg\;g^{-1}\right)$	203.87	162.60	116.48	65.31	34.48
	$q_{e,exp}\left(mg\;g^{-1}\right)$	203.69	162.23	116.28	65.14	34.46
	$n_{B}$	1.426	1.532	1.442	1.540	1.498
	$R_{adj}^{2}$	0.999	0.998	0.999	0.998	0.998

**Table 5:** Theoretically estimated column parameters of the Adams–Bohart, Thomas, and Yoon–Nelson models, along with estimated values for 2%, 5%, and 10% volume concentration for CO_2_ in the gas feed.

Model	Fixed Parameter	Value	Estimated Parameter	2 vol% CO_2_	5 vol% CO_2_	10 vol% CO_2_
Adams–Bohart	$h\left(cm\right)$	2.0	$k_{AB}\left(mL\;{mg}^{-1}\;{min}^{-1}\right)$	7.104	5.178	5.977
	$v(cm\;{min}^{-1})$	19.7	$N_{0}(mg\;{mL}^{-1})$	10.68	15.07	16.71
			$R_{adj}^{2}$	0.992	0.991	0.998
Thomas	W (g)	1.0	$k_{TH}\left(mL\;{mg}^{-1}\;{min}^{-1}\right)$	4.196	2.980	2.780
	$Q({cm}^{3}\;{min}^{-1})$	100.0	$q_{e,TH}(mg\;g^{-1})$	122.89	175.06	194.79
			$q_{e,exp}(mg\;g^{-1})$	141.79	182.48	203.69
			$R_{adj}^{2}$	0.998	0.999	0.996
Yoon–Nelson	-	-	$k_{YN}({min}^{-1})$	0.155	0.269	0.481
	-	-	τ(min)	33.28	19.40	11.23
			$R_{adj}^{2}$	0.998	0.999	0.996

**Table 6:** CO_2_ adsorption capacity values during the cyclic experiment (10 consecutive adsorption–desorption cycles).

Cycle Number	CO_2_ Adsorption Capacity (mg g^−1^)	Cycle Number	CO_2_ Adsorption Capacity (mg g^−1^)
1	204	6	200
2	202	7	202
3	202	8	203
4	200	9	205
5	205	10	203

## Data Availability

The data presented in this study are available on request from the corresponding author.
